# Consistent asymmetry in DNA damage artefacts across target regions in exome sequencing data

**DOI:** 10.1093/nargab/lqaf120

**Published:** 2025-08-27

**Authors:** Tyler D Medina, Declan Bennett, Cathal Seoighe

**Affiliations:** School of Mathematical and Statistical Sciences, University of Galway, University Road, Galway H91 TK33, Ireland; Research Ireland Centre for Research Training in Genomics Data Science, Research Ireland, Three Park Place, Hatch Street Upper, Dublin D02 FX65, Ireland; School of Mathematical and Statistical Sciences, University of Galway, University Road, Galway H91 TK33, Ireland; Department of Computational Biology, St. Jude Children’s Research Hospital, MS 1135, Room IA6038, 262 Danny Thomas Place, Memphis, Tennessee, 38105-3678, United States; School of Mathematical and Statistical Sciences, University of Galway, University Road, Galway H91 TK33, Ireland

## Abstract

Oxidative damage can introduce G>T mutations upon DNA replication. When this damage occurs *ex vivo*, sequenced DNA exhibits strand asymmetry, whereby sequence alignment yields G>T mismatches without corresponding C>A mismatches on the complementary strand at a given locus. Strand asymmetry is used to identify potential sequencing artefacts in somatic variant calls in cancer sequencing projects. Consistent with previous studies, we found that the strandedness of this asymmetry is frequently shared across targeted capture regions. However, while some exome sequencing datasets displayed consistent asymmetry relative to the forward reference strand, some surprisingly showed asymmetry relative to the transcription strand. Though oxidation is the principle cause of artefactual G>T mutations, we propose that the asymmetry stems from the use of single-stranded exome capture probes, as we did not find consistent asymmetry in matched whole genome sequencing. We further propose that high levels of asymmetry can be indicative of oxidation artefacts in the reported somatic variant calls of some samples. While most analysed cohorts showed low to moderate asymmetry, in one cohort (testicular germ cell tumour), approximately half of the reported G>T somatic mutations were likely to be oxidative damage artefacts, as indicated by the extent of asymmetry in mismatches and variants.

## Introduction

In paired-end next-generation sequencing (NGS), orientation bias is a phenomenon in which the evidence for an alternative allele at a given locus is asymmetrically present on forward- versus reverse-aligning DNA fragments. It is strongly associated with artefactual oxidative G>T mutations [1]. Such mutations arise through the oxidation of guanine to 8-oxo-guanine (8-oxo-G), followed first by replication of 8-oxo-G via non-canonical Hoogsteen base pairing between syn-8-oxo-G and adenine, rather than canonical pairing with cytosine, and subsequent replication of adenine to thymine [2, 3]. Due to this two-step mutation mechanism, 8-oxo-G mutations can show strong orientation bias when they occur *ex vivo* during sequencing library replication steps such as polymerase chain reaction (PCR): mutations are present only on the DNA fragments replicated from the original 8-oxo-G strand, but not on those of the complement strand. A similar mechanism is also seen in cytosine deamination artefacts, which commonly arise from formalin-fixed paraffin-embedded (FFPE) samples and display similar orientation bias [4–6].

For a given sequencing read on Illumina platforms, palindromic sequencing adapters make it possible to identify the parent and complement strands of the original double-stranded DNA (dsDNA) fragment after sequencing; coupled with the alignment direction of the reads, it is further possible to determine whether the parental strand belonged to the reference genome strand or the complement [7]. In the case of 8-oxo-G, true oxidative G>T mutations can occur *in vivo* as a result of oxidative stress, as seen in mutational signature SBS18 [8–10]. These *in vivo* mutations result in a mutated base pair, evidence for which propagates through both strands after adapter ligation and further library preparation, and thus no significant orientation bias is present [7]. When 8-oxo-G mutations instead display an asymmetric proportion of mismatches originating from the parent strand over the complement (or vice versa), this orientation bias is characteristic evidence of an 8-oxo-G *ex vivo* technical artefact. As a result, these mutations should not be considered to be true biological variants or somatic mutations [1, 7].

Somatic variant callers such as GATK Mutect2 and filtering tools such as SOBDetector use orientation bias to identify and remove G>T artefacts. This is primarily done by calculating the fraction of alternative-allele-supporting reads that are forward strand reads (reads with F1 and R2 orientation in paired-end sequencing) at a given putatively mutated site [1, 6, 11, 12]. Costello *et al.* and Chen *et al.* further demonstrated through their respective ArtQ and Global Imbalance Value (GIV) metrics that there is measurable orientation asymmetry across loci, suggesting that the direction of orientation bias can be conserved across loci in a sequencing sample [1, 7].

In this work, we further investigate G>T bias across loci. Using both tumour whole exome sequencing (WES) and whole genome sequencing (WGS) data from The Cancer Genome Atlas (TCGA), as well as germline WES data from the UK Biobank (UKB), we demonstrate that the characteristic asymmetric evidence for G>T artefacts can be consistently enriched on one DNA strand across loci in a sample. However, we further propose that the direction of this bias results from the use of single-stranded WES capture kits, such that the otherwise consistent direction of asymmetric enrichment can also reverse in accordance with the DNA strand that is targeted by the capture kit probes at that locus.

We suggest that the target strand be considered when filtering for G>T artefacts so as to increase the probability of removing true negative G>T variants that are consistent with the pattern of asymmetry by target strand, and reduce the probability of incorrectly removing true positives when the direction of asymmetry is inconsistent with this pattern.

As previously highlighted by Chen *et al.* in the TCGA lung adenocarcinoma cohort, we also point out that similar consistent asymmetry is found in the number of G>T versus C>A variant calls reported in other TCGA cohorts; in particular, the TCGA testicular germ cell tumour cohort displays a very high proportion of reported somatic mutations that are likely oxidative damage artefacts even after existing filtering methods have been applied.

## Materials and methods

### Whole exome and whole genome sequencing data

From all TCGA WES samples, we identified the most commonly used whole exome capture kit, NimbleGen SeqCap EZ HGSC VCRome v2.1 (catalogue ID: 06465668001), which was used in 1101 WES samples. Analysis was limited to TCGA cases that have both WES and WGS data for comparison, as well as Mutect2 variant calls, leaving 693 cases from 9 TCGA cohorts with WES, WGS, and variant call data. WGS samples were subset to chromosome 21 to reduce computation, and subset to the same capture kit regions as the exome samples.

From the UK Biobank, we analysed alignment files and plink2 [13] formatted genotypes from the October 2020 UKB release. From the 200 643 samples in the UKB release, we restricted analysis to individuals who self-identified as “White British” and who clustered as such in principal component analysis (PCA) to avoid population structure effects in variant analysis, leaving 165 519 individuals. For all analyses, data files were subset using the relevant sequencing capture kit, Integrated DNA Technologies (IDT) xGen Exome Hyb Panel v1. Genotype files were converted to multi-sample BCF format prior to analysis. Analysis of UKB samples was restricted only to G>T mismatches and variants due to cohort size and computation time required.

### Alignment mismatch analysis

To examine the presence of asymmetry in single-nucleotide alignment mismatches, we created a custom pipeline to count high-confidence mismatches to the reference genome. BAM files were primarily manipulated using Samtools [14], then post-processed and analysed with AWK and Python 3.

We first filtered alignments to remove lower quality reads, which included reads that were secondary, supplementary, or duplicate alignments; that were unmapped or had an unmapped mate; that had failed vendor quality control; or that had a mapping quality <60. We then subset these filtered BAM files using the NimbleGen VCRome capture kit, and split each file by coding strand direction, as annotated in the capture kit BED file. For each coding strand-specific BAM file, we used Samtools to produce pileup files, restricting pileup to bases with a base quality score of at least 37. Sites without mismatches were removed with AWK prior to parsing with Python. We then restricted analysis only to sites with a single mismatch to remove true biological variation and leave only mismatches most likely to be technical artefacts. Finally, we tallied each single-base mismatch type (C>T, G>A etc.) by coding direction per sample, then normalized each count by the nucleotide composition of the captured exome. These counts were used to test for two forms of mismatch incidence bias: asymmetry relative to the forward strand of the reference genome (which we refer to asymmetry by the reference strand), in which mismatches occur asymmetrically between the forward and reverse strands of the reference genome; and asymmetry by transcription strand, in which mismatches occur asymmetrically between the coding and template strands.

Asymmetry by reference strand of a given mismatch type (e.g. G>T) was calculated as the ratio of the number of mismatches of that type relative to the forward strand of the reference genome versus the number of mismatches against the reverse strand, or equivalently, versus the number of complementary mismatches (e.g. C>A) against the forward strand. Asymmetry by transcription strand of a given mismatch type was calculated similarly, but using the template strand of the gene at any given locus in place of the forward strand. By doing so, the asymmetry ratio was calculated as the number of mismatches against the template strand versus the number of mismatches against the coding strand, or equivalently, versus the number of complementary mismatches against the template strand.

### Variant call analysis

Variant calls were first filtered to separate tumour calls from matched normal calls (TCGA only) and to restrict to biallelic single-nucleotide variants (SNVs). We then tallied each call type by the coding direction annotated by the capture kit, while also including information on variant call filter status. For UKB samples, the same analysis was performed on germline variant calls after removing common SNPs as defined by gnomad [15], though no information on filter status was available in the data.

As in the alignment mismatch analysis, we calculated asymmetric incidence of variant versus complement, both by reference strand and by transcription strand. In addition, TCGA SNV counts were also compared by filter status, calculating asymmetry values both for all calls and for “PASS” calls only. Non-“PASS” variants in samples were filtered with GATK by their respective TCGA studies using 11 criteria, rejecting variant sites if they were found in normal tissue (alt_allele_in_normal, panel_of_normals, germline_risk), displayed strand bias (bPCR, bSeq, oxog), had multiple events (clustered_events, homologous_mapping_event, multi_event_alt_allele_in_normal, triallelic_site), or if evidence was not sufficient above background noise (t_lod_fstar). Variant call asymmetries were then also compared with the mismatch asymmetries per sample.

In addition to comparisons across samples, we also aggregated all unique SNV sites per TCGA cohort to eliminate, for example, the repeated measurement of shared driver mutations across samples of the same cancer type. We then evaluated departure from the expected binomial distribution of each SNV/complement pair, in which the expected binomial probability of each nucleotide was taken from the nucleotide composition of the captured regions of the reference genome.

### Somatic mutation rate estimation

We estimated the expected number of somatic mutations given the number of alignment mismatches in UKB samples (equation 1). The number of somatic mutations was estimated using an empirically derived equation based on age of the individual [16], from which we used the median somatic mutation load matching the age distribution of the UK biobank, yielding an estimated 2101 substitutions per cell. For genome size, we used the diploid size of the reference genome used in the UKB study, including only assembled chromosomes and unplaced contigs, totalling 6 185 543 820 bases. The number of sequenced bases was calculated as the mean number of sequenced reads per sample after filtering (44 487 029) multiplied by the read length used in the study (50 bp). For mismatches, we used the mean number of mismatches per sample after removing germline and low quality sites, leaving 173 327 mismatches per sample on average. Based on these variables, we estimated that on average 0.4% of mismatches are true somatic mutations.


(1) \begin{eqnarray*}
&& \frac{{\rm Somatic}\:{\rm mutations}}{{\rm Mismatch}} =\nonumber\\ && \frac{\frac{{\rm Somatic}\:{\rm mutations}}{bp}\:*\:{\rm Sequenced}\:{\rm bases}}{{\rm Mismatches}}
\end{eqnarray*}

## Results

### Asymmetry by reference strand in TCGA cancer cohorts

**Figure 1.**
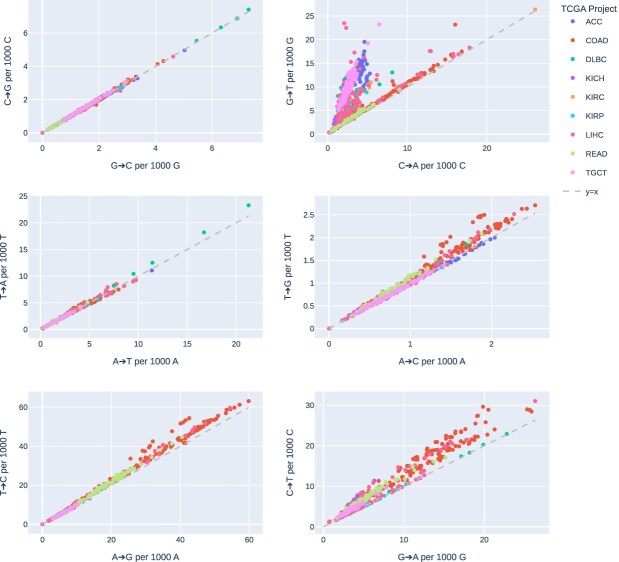
Mismatch versus complement asymmetry by reference strand in TCGA WES. The number of mismatches against the reference strand, versus the number of complement mismatches against the reference strand, per sample for each of the six possible mismatch/complement pairs, coloured by TCGA cohort. Departure from the dashed *y* = *x* line indicates asymmetric frequency of mismatch versus complement.

For each of the TCGA samples included in this study, we identified all loci for each sample at which there was a high-confidence base call differing from the reference genome. Within transversion mismatches, G>T not only predominated, comprising over half of all transversions but were also the third most common of any mismatch type overall, outnumbering both C>T and G>A transitions. Despite the high frequency of G>T mismatches, less than half as many C>A mismatches occurred overall. As such, G:C>T:A mismatches exhibited the greatest asymmetry between the forward and reverse strands of the reference genome with an overall median G>T/C>A ratio of 2.21 and no single sample having fewer G>T than C>A mismatches (Fig. 1). All other mismatch-versus-complement pairs exhibited median ratios between 1.01 and 1.03 overall, except for C>T/G>A at 1.26.

Across the TCGA cohorts, the per-cohort median ratio of G>T/C>A (relative to the forward strand of the reference genome) ranged from 1.11 and 1.14 [in rectal adenocarcinoma (READ) and colon adenocarcinoma (COAD), respectively] to 3.81 and 3.93 [in kidney renal clear cell carcinoma (KIRC) and testicular germ cell tumour (TGCT), respectively; Supplementary Fig. S1]. While COAD and READ exhibited low levels of G>T/C>A reference strand asymmetry compared to other cohorts, 27 out of the 197 COAD samples did have reference strand asymmetries >1.5, with eight samples >3.5. While colon and rectal adenocarcinomas share disease aetiologies which may suggest a biological origin for this pattern, similarities in reference strand asymmetry also appeared between samples from dissimilar cohorts sequenced together in the same time period. As all samples analysed were sequenced at the same facility, this indicates that these patterns are more likely related to tissue processing, sample handling, and sequencing protocols rather than disease biology (Supplementary Fig. S2). For cohorts containing samples that were sequenced months apart, samples generally form clusters of similar asymmetry values that can differ from clusters from the same cohort at different times. For example, although very few of the 197 COAD samples exhibited asymmetry, the only COAD samples sequenced in 2014 were the eight COAD samples with extreme asymmetry. These 8 samples were sequenced in the same 10-day period as 11 KIRC samples, some together on the same flowcells, with all 19 samples showing G>T/C>A reference asymmetry between 3.58 and 4.56.

Compared to the WES samples, the WGS samples showed far less evidence of asymmetry by reference strand and were more homogeneous across cohorts. G>T and C>A were also common in TCGA WGS samples, making up 29.17% of mismatches, and were second only to T>G / A>C mismatches (31.89%), which are known to be induced by the common chemotherapy drug 5-fluorouracil [17]. Excluding T>G / A>C, G>T and C>A were the most abundant mismatch pair in 96.63% of WGS samples. Transition mismatches were comparatively very low, accounting for only 8.88% of mismatches. For all mismatch/complement pairs, the distribution of all asymmetry by forward reference strand ratios for all WGS cohorts had medians between 0.91 and 1.03 (Supplementary Figs S4 and S6). While asymmetry by transcription strand was also minimal with cohort medians of 0.95–1.10, some T>any enrichment was also present, as in WES samples (Supplementary Figs S5 and S6). Despite this low asymmetry, we did note differences in forward- versus reverse-aligned mismatches. Across all mismatch types for all cohorts, the median ratio of forward- versus reverse-aligned mismatches typically ranged from ∼0.5 to 2.0. However, G>T and C>A showed particularly large differences between alignment directions, and were the most unbalanced mismatch type in 6 of 9 cohorts. G>T were consistently enriched on forward alignments, while C>A were consistently enriched on reverse alignments, reaching 4*x*–6*x* enrichment in some cohorts. This relationship was not observed in TCGA WES data, in which mismatch alignment directions were generally well-balanced.

**Figure 2.**
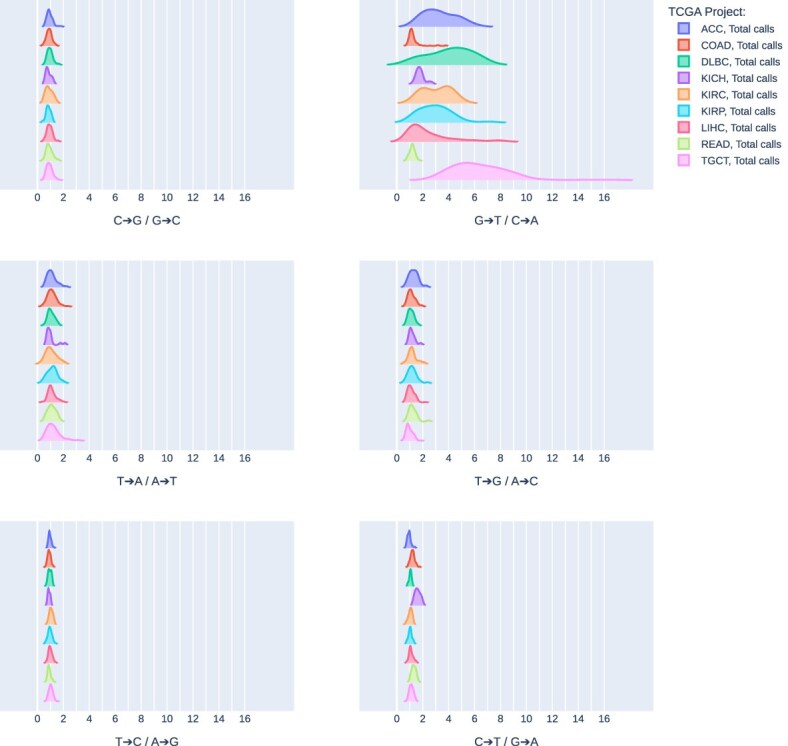
Variant calls versus complement asymmetry by reference strand in TCGA WES. Distribution of ratios of variants versus variant complements, relative to the reference strand, per TCGA cohort for each of the six possible variant/complement pairs, coloured by cohort. All unfiltered variant calls are included.

**Figure 3.**
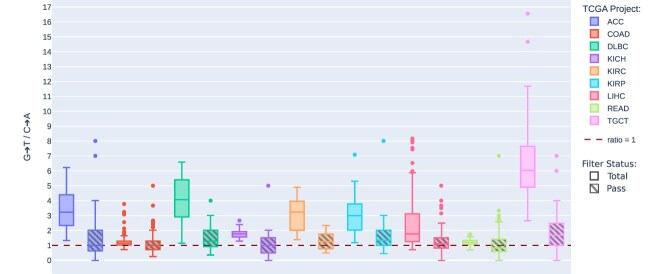
G>T/C>A variant call ratios by reference strand in TCGA WES. The distribution of G>T versus C>A variant call ratios, relative to the reference strand, for nine TCGA cohorts. Unfiltered (solid fill) and post-filtering (striped fill) variants are shown. The dashed red line indicates the ratio = 1 line, corresponding to equal presence of G>T and C>A variants.

In the unfiltered set of putative somatic variants identified in the TCGA samples (prior to filtering), patterns of G>T/C>A variant call asymmetry by reference strand were similar to the patterns of G>T/C>A mismatch asymmetry by reference strand in the same samples (Fig. 2). Median call-versus-complement ratios of each mutation type were found to vary between 0.899 and 1.10, except for G>T/C>A at 1.78. Per cohort, ratios varied similarly, with all non-G>T/C>A calls found with median asymmetry ratios between 0.810 and 1.56. G>T/C>A ratios varied considerably by cohort. Similarly for mismatch asymmetry, COAD and READ displayed the lowest median asymmetry by reference strand for total called variants (1.14 and 1.19, respectively), while TGCT displayed the highest (5.87). After filtering (see “Materials and methods” section, Variant call analysis), remaining “PASS” variants generally had greatly reduced asymmetry, though some cohorts, including TGCT, still retained elevated levels of asymmetry post-filtering (Fig. 3).

**Figure 4.**
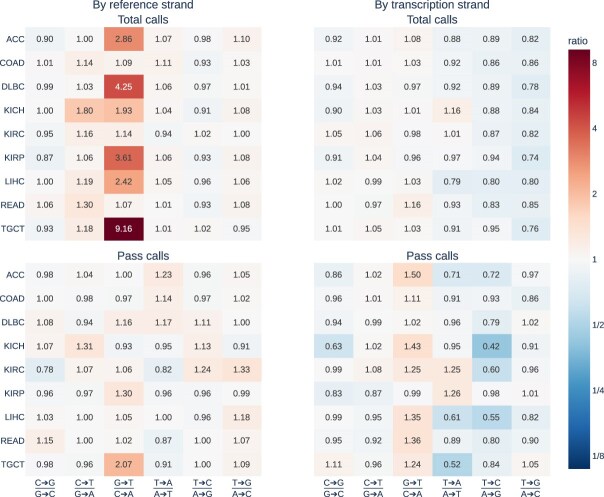
Unique variant call sites versus complements in TCGA WES. Median variant call versus complement ratios for each variant/complement pair for each TCGA cohort. Unfiltered variant ratios are shown at top, and post-filtering variants are shown at bottom. Ratios relative to the reference strand are show at left, and ratios relative to the transcribed strand are shown at right.

Of particular note, 9 times as many G>T versus C>A variants (relative to the forward strand of the reference genome) were called in the TGCT cohort in aggregate prior to filtering. Even after filtering, over twice as many G>T mutations were called compared to the complementary C>A mutations (403 versus 195). No other cohort showed total or filter-passing call asymmetry by reference strand as extreme (Fig. 4). Of the 403 unique G>T variant sites that passed filtering in the TGCT cohort, the most common flanking nucleotide context was GGC (39, 9.68%), which has previously been shown to be one of the most frequently oxidised G nucleotide contexts [18]. The GGC context was less common in variants that pass filtration from other cohorts (1.65%–5.03%), with the exception of the LIHC cohort (151/1658, 9.11%). Notably, LIHC and TGCT alone each show marginally significant correlation between the G>T/C>A mismatch ratio and pass variant call ratio by reference strand (Pearson correlation = 0.295 and 0.212, *P* = 9.75e-4 and 4.46e-2, respectively).

Asymmetry by transcription strand was far less present in TCGA WES samples. No mismatch/complement pair displayed asymmetry by transcription strand as high as G>T/C>A reference asymmetry. However, T>N mismatches were consistently found more often than A>N mismatches regardless of mutation type by ∼10%, with a consistent pattern of relative differences between cohorts conserved across T>N mutation types (Supplementary Fig. S3). Total WES variant calls likewise did not demonstrate extreme asymmetry by transcription strand, with median overall ratios per mutation type ranging from 0.83 to 1.35 and with little variation between cohorts (Supplementary Fig. S7). Additionally, among total unique sites at which variants were called, no mutation type exhibited extreme asymmetry in any cohort, with all ratios lying between 0.74 and 1.16.

### Asymmetry by transcription strand in UKB germline samples

**Figure 5.**
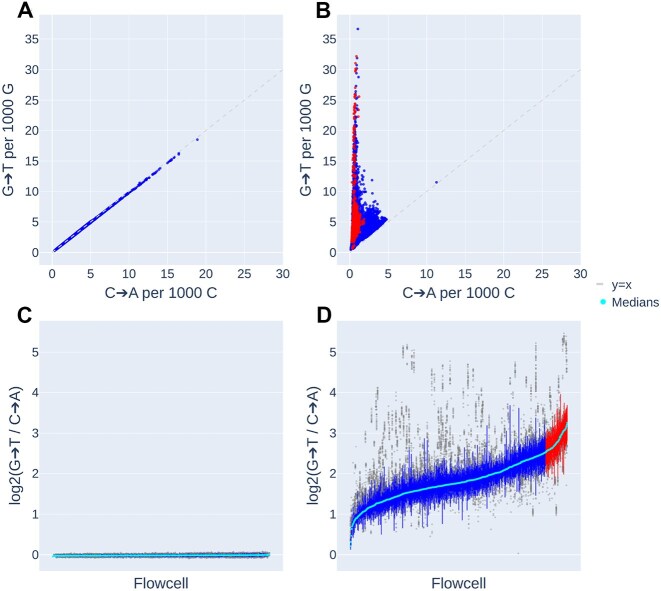
G>T/C>A mismatch versus complement asymmetry in UKB WES. Top: Number of G>T versus C>A mismatches in the UKB, relative to the (**A**) reference strand and (**B**) transcribed strand. Bottom: Distribution of G>T versus C>A mismatch ratios, log_2_ transformed, per sequencing flowcell, relative to the (**C**) reference strand and (**D**) transcribed strand. Outliers per flowcell are shown in grey. Median per flowcell is shown in cyan. Flowcells with the highest 10% median ratios are highlighted in red below, with samples from those flowcells highlighted in red at top.

In contrast to the TCGA, samples in the UK Biobank demonstrated very little evidence of asymmetry between the forward and reverses strand of the reference genome (median ratio = 0.990, SD = 1.16e-2) (Fig. 5A and C). We obtained similar results for G>T reference strand asymmetry in UKB heterozygous SNVs (median ratio = 0.912, SD = 5.34e-2; Supplementary Fig. S8A) and homozygous SNVs (median ratio = 0.898, SD = 5.77e-2).

Surprisingly, we found that asymmetry in the UKB samples was much more pronounced and variable when calculated with respect to the transcription strand, with a median G>T versus C>A mismatch asymmetry ratio across all samples of 3.42 (SD = 2.09; Fig. 5B and D). Over a third of all samples (60 421) showed G>T versus C>A ratios >4.0, 265 of which were found to have extreme ratios between 20.0 and a maximum of 44.02. No single sample was found to have fewer C>A than G>T mismatches. Like TCGA samples, the extent of asymmetry appears to be batch-dependent, such that samples sequenced together on the same flowcell showed similar asymmetries (Fig. 5D). Variant calls, however, showed far less evidence of asymmetry by transcription strand, with asymmetry ratios ranging from 0.730 to 1.23 for heterozygous SNVs (median ratio = 0.953, SD = 5.59e-2) (Supplementary Fig. S8B) and from 0.650 to 1.21 for homozygous SNVs (median ratio = 0.869, SD = 5.64e-2). Among the called variants there were, in fact, ∼6% (4.25M) fewer G>T than C>A calls made in aggregate.

## Discussion

Both tumour data from TCGA and germline data from the UKB show evidence of asymmetry that is consistent with respect to DNA strand, particularly in the numbers of G>T single-nucleotide mismatches found per sample, though in different ways. Limited evidence of C>T asymmetry was also noted, and likely arose from cytosine deamination artefacts, which are similar in nature to 8-oxo-G artefacts [4, 6]. However, cytosine deamination is generally associated with FFPE tissue, and as none of the TCGA WES samples we analysed were formalin-fixed, low incidence of cytosine deamination artefacts compared to 8-oxo-G artefacts is to be expected [4, 5].

All UKB samples consistently show an unexpected bias towards G>T mismatches on the template strand of the targeted exonic regions. While consistent strand asymmetry relative to the forward strand of the reference genome in TCGA cohorts has been described previously by various metrics [1, 7], asymmetry by transcription strand has not been reported to the best of our knowledge. Furthermore, because these technical artefacts reflect the biological pattern of transcription, they may also confound efforts to investigate mechanisms such as transcription-associated DNA damage [19, 20]. While transcription damage may contribute to the patterns of asymmetry we observed, this would require the observed G:C>T:A mismatches to be largely due to somatic mutations. However, based on the number of somatic mutations expected in a 56-year-old individual (the mean age of UKB samples) and the mean number of mismatches per UKB sample, we estimate that only 0.4% of mismatches are likely to be somatic mutations (see “Materials and methods”: Somatic mutation rate estimation) [16]. This indicates that true somatic mutations, such as from transcription-associated DNA damage, cannot explain the high asymmetry by transcription strand. Furthermore, the level of asymmetry by transcription strand in UKB samples varies substantially across sample batches, indicating that these mismatches are technical artefacts and not somatic mutations.

We propose that the asymmetry in both UKB and TCGA samples are not distinct phenomena, but that both arise from the same technical source, namely the use of single-stranded DNA (ssDNA) capture probes. ssDNA probes target either the forward or reverse strand, but not both strands at the same locus [21]. As a result, the selection of targets in an ssDNA capture kit can introduce a technical bias that appears biological if, for example, all probes are designed to target the known coding sequences of all genes. Of the 19 398 genes targeted by the UKB capture kit (IDT xGen Exome Hyb Panel v1), 18 985 are known Ensembl-annotated genes. According to Ensembl release 113 and information obtained via correspondence with IDT, of these genes, all but 7 (>99.9%) were consistently targeted with probes complementary to the template strand of the gene. This results in the capture of 8-oxo-G DNA damage on the template strand, but not the coding strand, resulting in an enrichment of coding strand C>A mutations. Per correspondence with Roche (the owners of the capture kit used in the TCGA samples, NimbleGen SeqCap EZ HGSC VCRome v2.1), the TCGA capture kit probes were designed against the forward strand of the reference genome only. By the same mechanism, this produces an excess of G>T versus C>A mismatches relative to the forward strand. With both capture kits, 8-oxo-G present on the captured strand leads to an apparent enrichment of G>T mutations on the captured strand, while any 8-oxo-G nucleotides present in the non-captured strand are lost and do not produce G>T mutations. This same phenomenon presumably occurs with any ssDNA capture kit, though remarkably, information on the precise nature of the probes used in these capture kits is not clearly provided in their technical specifications, despite the fact that this could be useful for identifying samples with high levels of DNA damage and for reducing the number of incorrectly called somatic variants. Of the two designs, we posit that capture kits designed to consistently target coding sequences can produce 8-oxo-G artefacts that problematically resemble biological processes, while capture kits designed to consistently target a reference genome strand do not.

The absence of strand-specific asymmetry in TCGA WGS samples, which by nature do not use a capture kit, lends further evidence to the role of WES capture kits in producing asymmetry. As noted by Costello *et al.*, differences in library preparation between WGS and WES could have limited the production of 8-oxo-G in the WGS samples [1], precluding the detection of any 8-oxo-G asymmetry that would provide evidence for a mechanism other than ssDNA capture kits. However, WGS samples did display enrichment of forward-aligning G>T and reverse-aligning C>T mismatches, suggesting that 8-oxo-G damage is present, yet without capture-induced strand asymmetry. Some TCGA cohorts showed similarly low asymmetry with high rates of G>T and C>A mismatches (e.g. TCGA-COAD). This could potentially be attributed to the time point at which 8-oxo-G damage is replicated to produce C>A mutations, which informs whether asymmetry will be detected: 8-oxo-G mutations produced prior to ssDNA capture (e.g. during whole genome library PCR) could produce G>T and C>A mutations on either strand symmetrically; 8-oxo-G mutations that occur post-capture (e.g. during whole exome amplification) would produce G>T mutations asymmetrically from captured oxidised fragments only. If, for example, the majority of TCGA-COAD samples underwent heavy oxidation predominantly before exome capture, many G>T/C>A mutations could be produced without introducing asymmetry. Across TCGA cohorts, the extent of mismatch asymmetry is similar between samples sequenced together in the same time period, possibly suggesting shared *in vitro* sources of 8-oxo-G production, such as reagent contamination [1].

The variant call asymmetry present in some samples suggests the need to factor the targeted strand into somatic variant calling tools. Current filtering methods do reduce asymmetry present in somatic variant calls, as seen across TCGA cohorts (Fig. 3); however, the TCGA-TGCT cohort also shows that asymmetry is not completely removed, as more than twice as many G>T versus C>A variants were identified, even after filtering in that cohort. Coupled with the relatively high proportion of GGC nucleotide contexts, this indicates that approximately half of the reported G>T calls in the TGCT cohort are likely to be the result of 8-oxo-G DNA damage. These findings are similar to those reported by Chen *et al.* in TCGA lung adenocarcinoma WES samples [7], though Stewart *et al.* state that the influence of 8-oxo-G DNA damage on somatic variant calls in this cohort is unlikely to result in such large numbers of artefact variant calls given the proportion of 8-oxo-G-associated nucleotide contexts versus the background non-8-oxo-G>T mutation rate [1, 22]. However, while certain nucleotide contexts have been shown to be readily oxidized to 8-oxo-G [18], and more 8-oxo-G artefacts are expected to be found at such sites, whether or not 8-oxo-G is more readily paired with adenine at particular contexts is less clear [23]. As such, we do expect 8-oxo-G artefacts to be over-represented by certain nucleotide contexts, but do not expect these artefacts to be restricted solely to these sites.

Previous studies have shown CGG to be the nucleotide context most associated with 8-oxo-G artefacts, and suggest that this is supported by Margolin *et al.*, who demonstrate that CGG is the G nucleotide context most amenable to oxidation [1, 22]. However, Margolin *et al.* also clearly demonstrate that the reactivity of G nucleotide contexts differs by the oxidant used. While CGG was shown to be the nucleotide context most reactive to riboflavin-mediated photo-oxidation (∼2.5 times as reactive as the GGC nucleotide context highlighted in our results), GGC was shown to be ∼7 times more reactive than CGG to oxidation by nitrosoperoxycarbonate [18]. Nitrosoperoxycarbonate is a known reactive product of inflammation [24, 25], and as such may be more representative of oxidation that occurs to a sample *in vivo*; photo-oxidation is perhaps more likely to occur *ex vivo* in DNA samples. The mechanism of oxidation in Costello *et al.* was reported to arise during acoustic shearing, potentially as a result of metal contamination [1]. With the variety of potential sources of oxidation and the differences in their capacity to oxidize different nucleotide contexts, we again suggest that nucleotide context is not a strict discriminator for 8-oxo-G artefacts, and that the levels of asymmetry seen in the TCGA-TGCT G>T versus C>A mismatches are large enough to affect variant calls.

In updated studies of TCGA, variant call asymmetry was also noted in samples originating from one specific sequencing center. This was addressed by implementing a “StrandBias” filter that removed excess G>T calls, beginning with those of lowest quality and continuing until call asymmetry was eliminated in samples from this center [26]. As demonstrated by this updated study, variant call asymmetry is a recognized problem. Though TCGA variant calls generally do include many low quality candidate variants that may be easily ignored in clinical analysis, or may be summarily removed as in Ellrott *et al.* [26], we caution that 8-oxo-G artefacts are likely to survive some filtration methods. We further note that the TCGA–TGCT cohort was not sequenced at the center targeted by the StrandBias filter in Ellrott *et al.* [27].

When we apply a similar filtering method, we find that the remaining higher quality G>T variants in the TGCT each demonstrate a much more balanced ratio of forward- versus reverse-aligning DNA fragments. This further suggests that 8-oxo-G artefacts are present in the lower quality G>T variants, and that they are affected by the high levels of G>T versus C>A mismatch asymmetry noted in the cohort. In addition, we also warn that very few passing G:C>T:A variants were reported per sample in the TGCT cohort, such that variant call asymmetry is not always evident per sample, but only becomes apparent in aggregate.

While the effect of target strand asymmetry is evident and has some impact on somatic variant calling, variant call asymmetry appears less frequently in UKB variant calling compared to TCGA. This is likely due to the differences in the goals and evidence used in cancer somatic variant calling versus population germline variant calling: somatic variant calling generally aims to find *de novo* variants, including clinically relevant low-frequency mutations in subclonal cancer cells, and so artefacts are more easily encountered and included among very low allele frequency mutations in the deeper sequencing that is typically employed; germline variant calling instead aims to establish genotypes based on comparatively high variant allele frequencies in lower depth of coverage sequencing, and thus can ignore such artefacts based on variant allele frequency alone.

To counter the effects of target strand asymmetry, we suggest that capture kit design and strandedness be factored into variant calling pipelines. Filtering methods such as strand odds ratios evaluate asymmetry per site [11, 12]. By additionally including prior knowledge of consistent target strand bias, G:C>T:A sites with strand asymmetry consistent with the capture strand bias direction should then require less evidence to be considered artefactual. Other metrics, such as ArtQ [1], do evaluate exome-wide asymmetry to assess the extent of 8-oxo-G artefact presence in a sample, but also fail to include the prior probabilities informed by the systematic variation in the direction of the asymmetry. As demonstrated by the UKB samples, asymmetry between the forward and reverse strands of the reference genome is effectively hidden exome-wide by the alternating direction of asymmetry according to coding orientation: while the whole exome does in effect demonstrate asymmetry by reference strand, half of the exome shows enriched G>T, while the other half shows enriched C>A, summing to neutrality exome-wide. Similarly, if the UKB capture kit had been used in the TCGA studies, measures such as ArtQ would fail to detect systematic strand bias. While Mutect2’s LearnOrientationBiasModel uses machine learning to assign prior probabilities of strand artefacts across all sites [12], we suggest that it too could benefit from the additional knowledge provided by capture kit design and the expected direction of bias at a given site. In summary, we propose the need to include the orientation of ssDNA capture probes in variant calling filtering algorithms to more accurately reduce the influence of 8-oxo-G artefacts in exome sequencing data, particularly due to the effect these artefacts can have on somatic variant calling and reporting.

## Supplementary Material

lqaf120_Supplemental_File

## Data Availability

This study is based on data generated by The Cancer Genome Atlas, which is accessible through the National Cancer Institute GDC Data Portal at https://portal.gdc.cancer.gov/. This study is also based on data generated by the UK Biobank, which is available through application to https://www.ukbiobank.ac.uk/. Source code used to generate and analyse data for this work is available on GitHub at https://github.com/TDMedina/StrandBias, and is archived on Zenodo at https://doi.org/10.5281/zenodo.14673618 and Figshare at https://doi.org/10.6084/m9.figshare.28219688.v1.

## References

[1] Costello M, Pugh TJ, Fennell TJ et al. Discovery and characterization of artifactual mutations in deep coverage targeted capture sequencing data due to oxidative DNA damage during sample preparation. Nucleic Acids Res. 2013; 41:e67. 23303777 10.1093/nar/gks1443 PMC3616734

[2] Beard WA, Batra VK, Wilson SH DNA polymerase structure-based insight on the mutagenic properties of 8-oxoguanine. Mutat Res. 2010; 703:18–23. 20696268 10.1016/j.mrgentox.2010.07.013 PMC3023916

[3] McAuley-Hecht KE, Leonard GA, Gibson NJ et al. Crystal structure of a DNA duplex containing 8-hydroxydeoxyguanine-adenine base pairs. Biochemistry. 1994; 33:10266–70. 8068665 10.1021/bi00200a006

[4] Hsu CW, Sowers ML, Baljinnyam T et al. Measurement of deaminated cytosine adducts in DNA using a novel hybrid thymine DNA glycosylase. J Biol Chem. 2022; 298:101638 10.1016/j.jbc.2022.101638. 35085553 PMC8861164

[5] Guo Q, Lakatos E, Bakir IA et al. The mutational signatures of formalin fixation on the human genome. Nat Commun. 2022; 13:4487 10.1038/s41467-022-32041-5. 36068219 PMC9448750

[6] Diossy M, Sztupinszki Z, Krzystanek M et al. Strand Orientation Bias Detector to determine the probability of FFPE sequencing artifacts. Brief Bioinform. 2021; 22:bbab186 10.1093/bib/bbab186. 34015811

[7] Chen L, Liu P, Evans TC Jr et al. DNA damage is a pervasive cause of sequencing errors, directly confounding variant identification. Science. 2017; 355:752–6. 28209900 10.1126/science.aai8690

[8] Markkanen E, Hübscher U, van Loon B Regulation of oxidative DNA damage repair: the adenine:8-oxo-guanine problem. Cell Cycle. 2012; 11:1070–5. 10.4161/cc.11.6.19448. 22370481

[9] Kawanishi S, Hiraku Y, Oikawa S Mechanism of guanine-specific DNA damage by oxidative stress and its role in carcinogenesis and aging. Mutat Res. 2001; 488:65–76. 10.1016/s1383-5742(00)00059-4. 11223405

[10] Alexandrov LB, Nik-Zainal S, Wedge DC et al. Signatures of mutational processes in human cancer. Nature. 2013; 500:415–21. 10.1038/nature12477. 23945592 PMC3776390

[11] Poplin R, Ruano-Rubio V, DePristo MA et al. Scaling accurate genetic variant discovery to tens of thousands of samples. bioRxiv24 July 2018, preprint: not peer reviewed 10.1101/201178.

[12] Benjamin D, Sato T, Cibulskis K et al. Calling Somatic SNVs and Indels with Mutect2. bioRxiv2 December 2019, preprint: not peer reviewed 10.1101/861054.

[13] Chang CC, Chow CC, Tellier LC et al. Second-generation PLINK: rising to the challenge of larger and richer datasets. Gigascience. 2015; 4:7. 25722852 10.1186/s13742-015-0047-8 PMC4342193

[14] Danecek P, Bonfield JK, Liddle J et al. Twelve years of SAMtools and BCFtools. GigaScience. 2021; 10:giab008 10.1093/gigascience/giab008. 33590861 PMC7931819

[15] Chen S, Francioli LC, Goodrich JK et al. A genomic mutational constraint map using variation in 76,156 human genomes. Nature. 2023; 625:92–100. 10.1038/s41586-023-06045-0. 38057664 PMC11629659

[16] Zhang L, Dong X, Lee M et al. Single-cell whole-genome sequencing reveals the functional landscape of somatic mutations in B lymphocytes across the human lifespan. Proc Natl Acad Sci USA. 2019; 116:9014–9. 10.1073/pnas.1902510116. 30992375 PMC6500118

[17] Christensen S, Van der Roest B, Besselink N et al. 5-Fluorouracil treatment induces characteristic T>G mutations in human cancer. Nat Commun. 2019; 10:4571 10.1038/s41467-019-12594-8. 31594944 PMC6783534

[18] Margolin Y, Shafirovich V, Geacintov NE et al. DNA sequence context as a determinant of the quantity and chemistry of guanine oxidation produced by hydroxyl radicals and one-electron oxidants. J Biol Chem. 2008; 283:35569–78. 10.1074/jbc.m806809200. 18948263 PMC2602890

[19] Lans H, Hoeijmakers JHJ, Vermeulen W et al. The DNA damage response to transcription stress. Nat Rev Mol Cell Biol. 2019; 20:766–84. 10.1038/s41580-019-0169-4. 31558824

[20] Milano L, Gautam A, Caldecott KW DNA damage and transcription stress. Mol Cell. 2024; 84:70–9. 10.1016/j.molcel.2023.11.014. 38103560

[21] Farrell RE Jr Chapter 16 - Nucleic Acid Probe Technology. RNA Methodologies. 2017; (5th edition) Academic Press 469–526. 10.1016/b978-0-12-804678-4.00016-6.

[22] Stewart C, Leshchiner I, Hess J et al. Comment on ‘DNA damage is a pervasive cause of sequencing errors, directly confounding variant identification’. Science. 2018; 361:eaas9824 10.1126/science.aas9824. 30262473 PMC7350422

[23] Ovcherenko SS, Shernyukov AV, Nasonov DM et al. Dynamics of 8-oxoguanine in DNA: decisive effects of base pairing and nucleotide context. J Am Chem Soc. 2023; 145:5613–7. 10.1021/jacs.2c11230. 36867834

[24] Margolin Y, Cloutier JF, Shafirovich V et al. Paradoxical hotspots for guanine oxidation by a chemical mediator of inflammation. Nat Chem Biol. 2006; 2:365–6. 10.1038/nchembio796. 16751762

[25] Dedon PC, Tannenbaum SR Reactive nitrogen species in the chemical biology of inflammation. Archiv Biochem Biophys. 2004; 423:12–22. 10.1016/j.abb.2003.12.017. 14989259

[26] Ellrott K, Bailey MH, Saksena G et al. Scalable open science approach for mutation calling of tumor exomes using multiple genomic pipelines. Cell Syst. 2018; 6:271–81. 10.1016/j.cels.2018.03.002. 29596782 PMC6075717

[27] Shen H, Shih J, Hollern DP et al. Integrated molecular characterization of testicular germ cell tumors. Cell Rep. 2018; 23:3392–406. 10.1016/j.celrep.2018.05.039. 29898407 PMC6075738

