# Supplementary material for: Consistent asymmetry in DNA damage artefacts across target regions in exome sequencing data
Source: NAR Genom Bioinform. 2025 Aug 27;7(3):lqaf120. doi: 10.1093/nargab/lqaf120 (PMC12390751; doi:10.1093/nargab/lqaf120)
Supplement: lqaf120_Supplemental_File [file lqaf120_supplemental_file.pdf]

Supplementary Figures

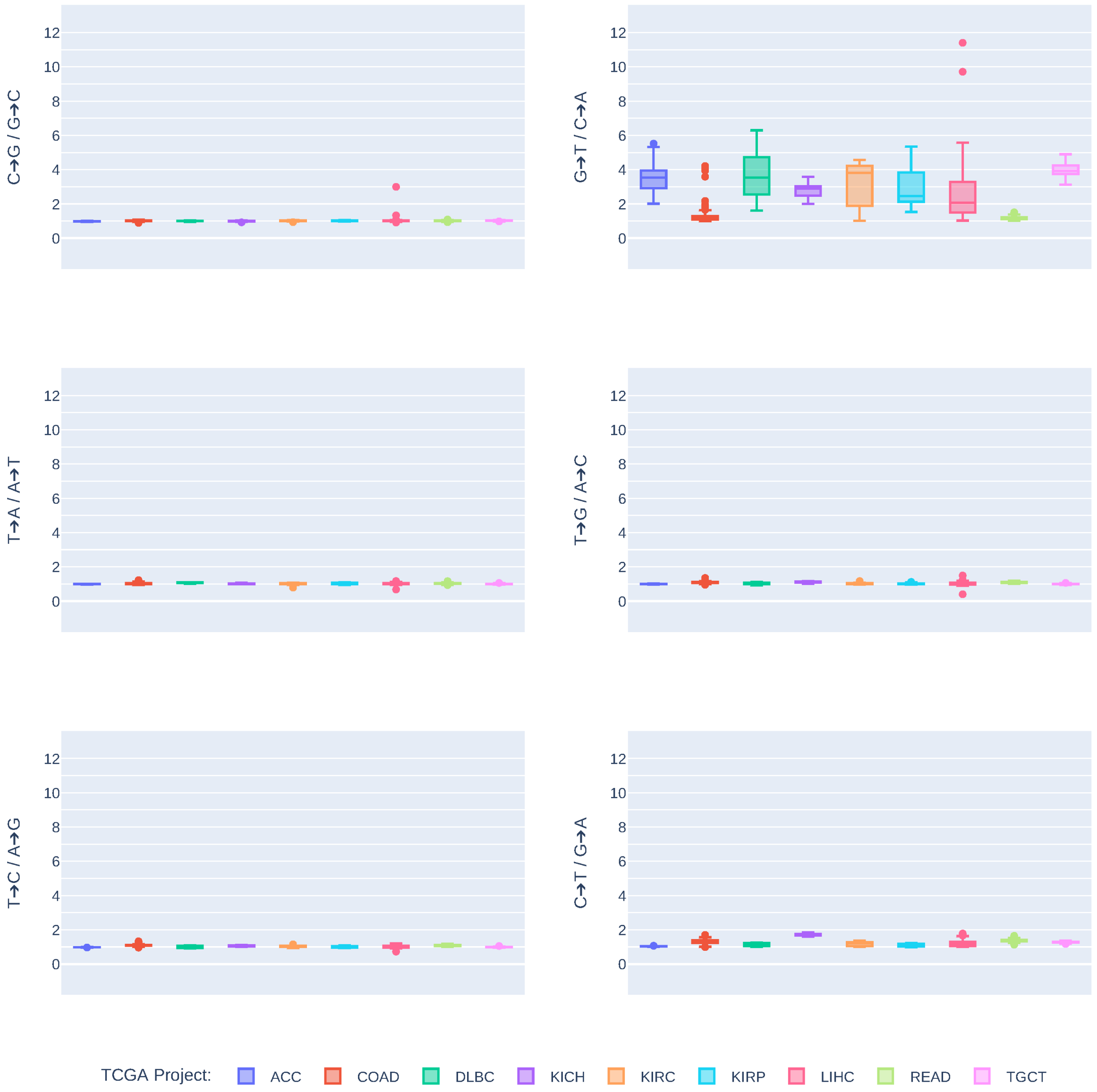

Fig. S1. Mismatch vs. complement asymmetry by reference strand in TCGA WES. Distribution of ratios of mismatches against the reference strand versus the number of complement mismatches against the reference strand, per cohort for each of the six possible mismatch/complement pairs.

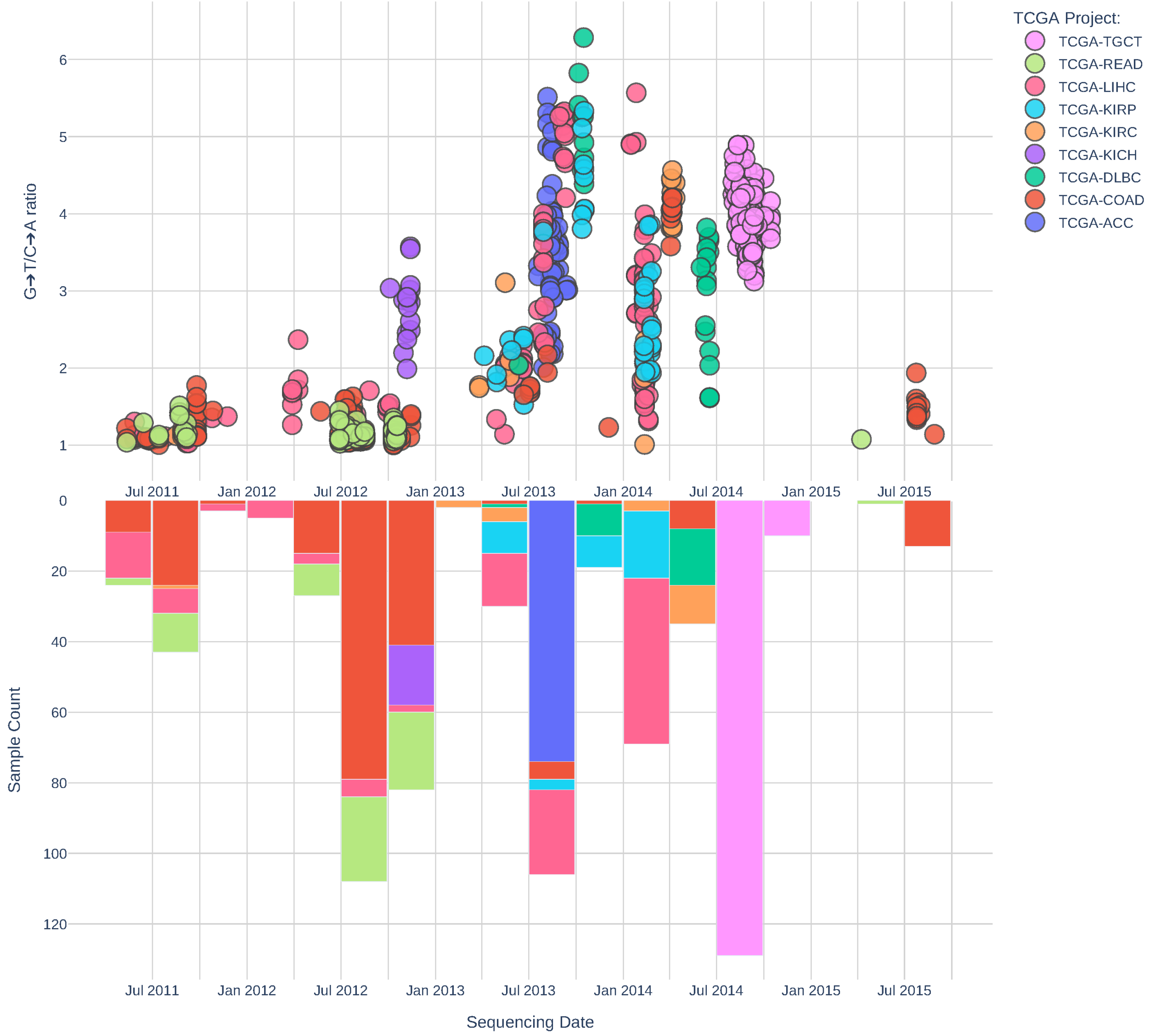

Fig. S2. G>T vs. C>A asymmetry by reference strand, by sequencing date in TCGA WES. *Top:* The ratio of G>T versus C>A mismatches against the reference strand is shown by sequencing date, as recorded in the metadata of the sequencing alignment file for the sample. Two extreme outlier LIHC samples (G>T/C>A ratios of 9.70 and 11.39 sequenced January 2014) omitted for visibility. *Bottom:* The number of samples sequenced in each 3-month period.

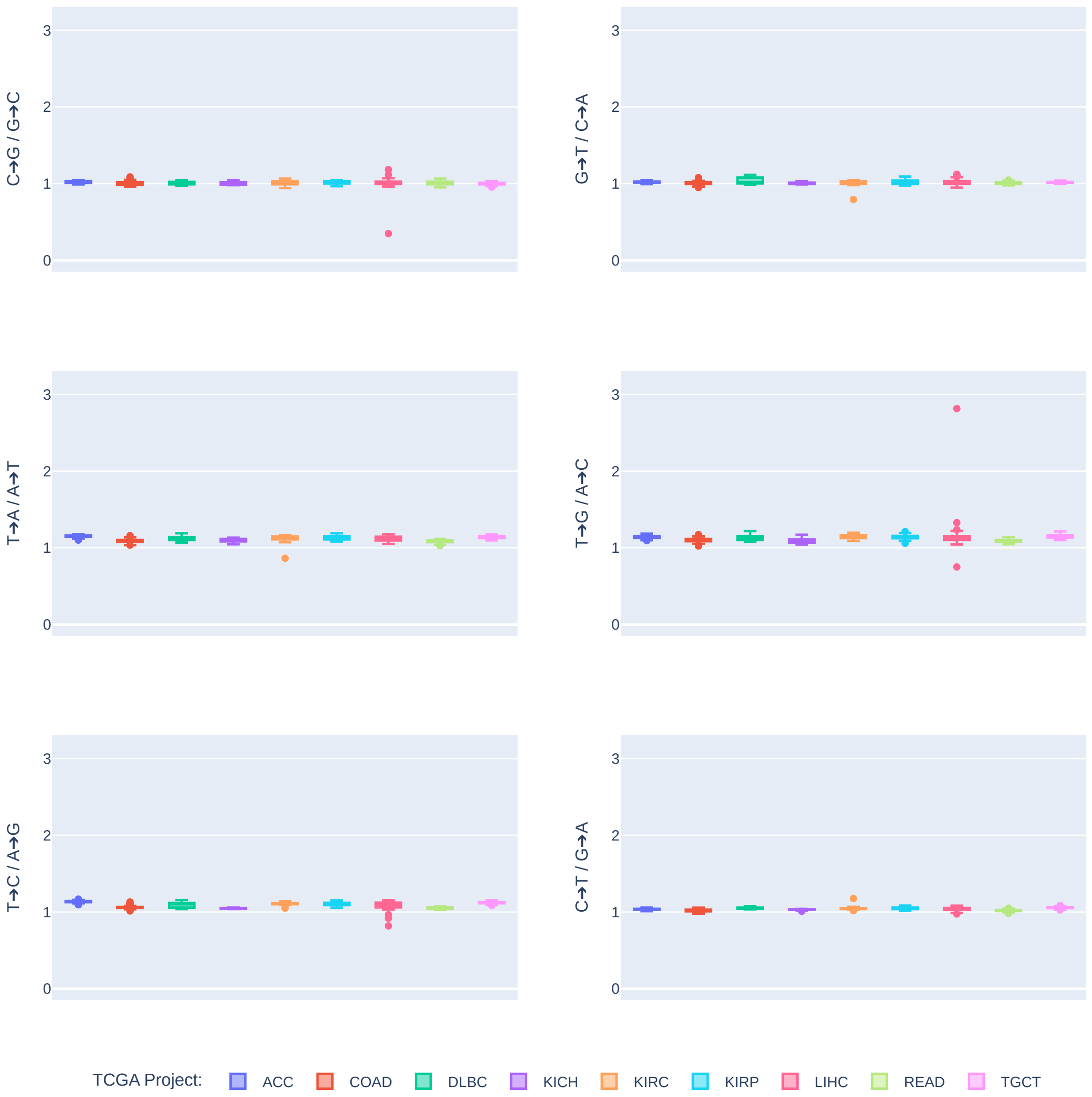

Fig. S3. Mismatch vs. complement asymmetry by transcription strand in TCGA WES. Distribution of ratios of mismatches against the transcribed strand versus the number of complement mismatches against the transcribed strand, per cohort for each of the six possible mismatch/complement pairs.

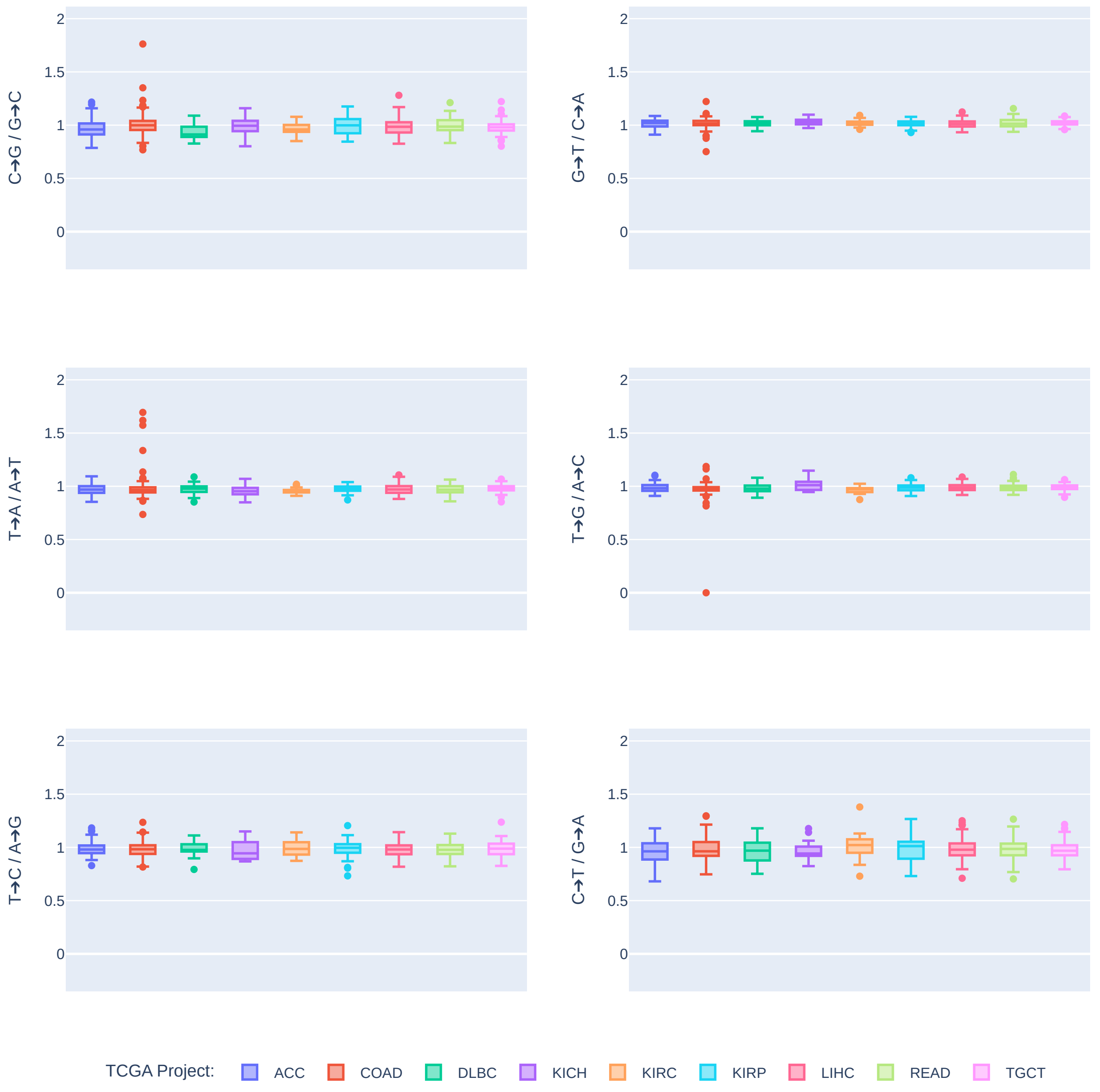

Fig. S4. Mismatch vs. complement asymmetry by reference strand in TCGA WGS. Figure depicts whole genome sequencing samples subset to chromosome 21. Subplots display the distribution of ratios of mismatches against the reference strand versus the number of complement mismatches against the reference strand, per cohort for each of the six possible mismatch/complement pairs.

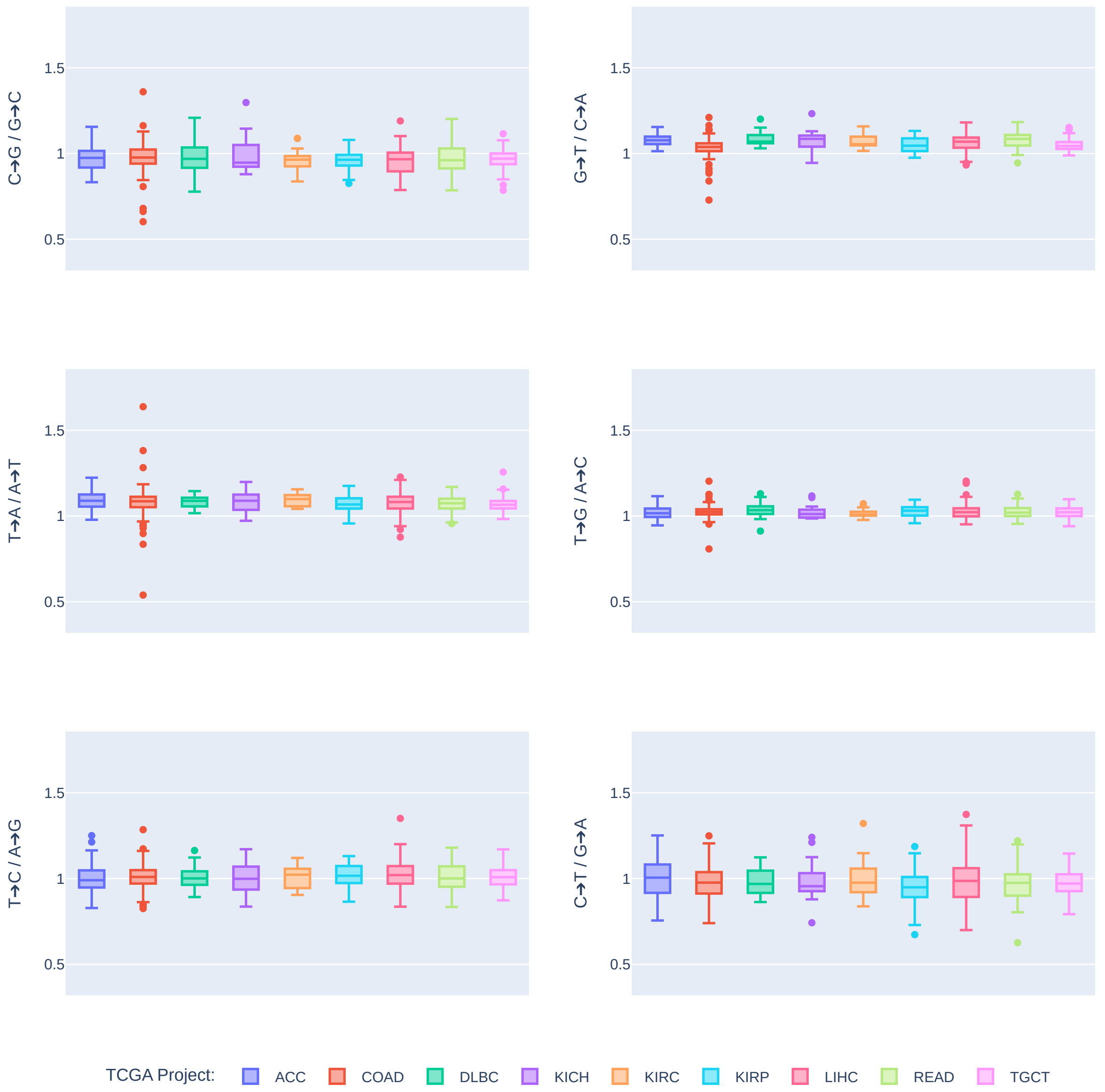

Fig. S5. Mismatch vs. complement asymmetry by transcription strand in TCGA WGS. Figure depicts whole genome sequencing samples subset to chromosome 21. Subplots display the distribution of ratios of mismatches against the transcribed strand versus the number of complement mismatches against the transcribed strand, per cohort for each of the six possible mismatch/complement pairs.

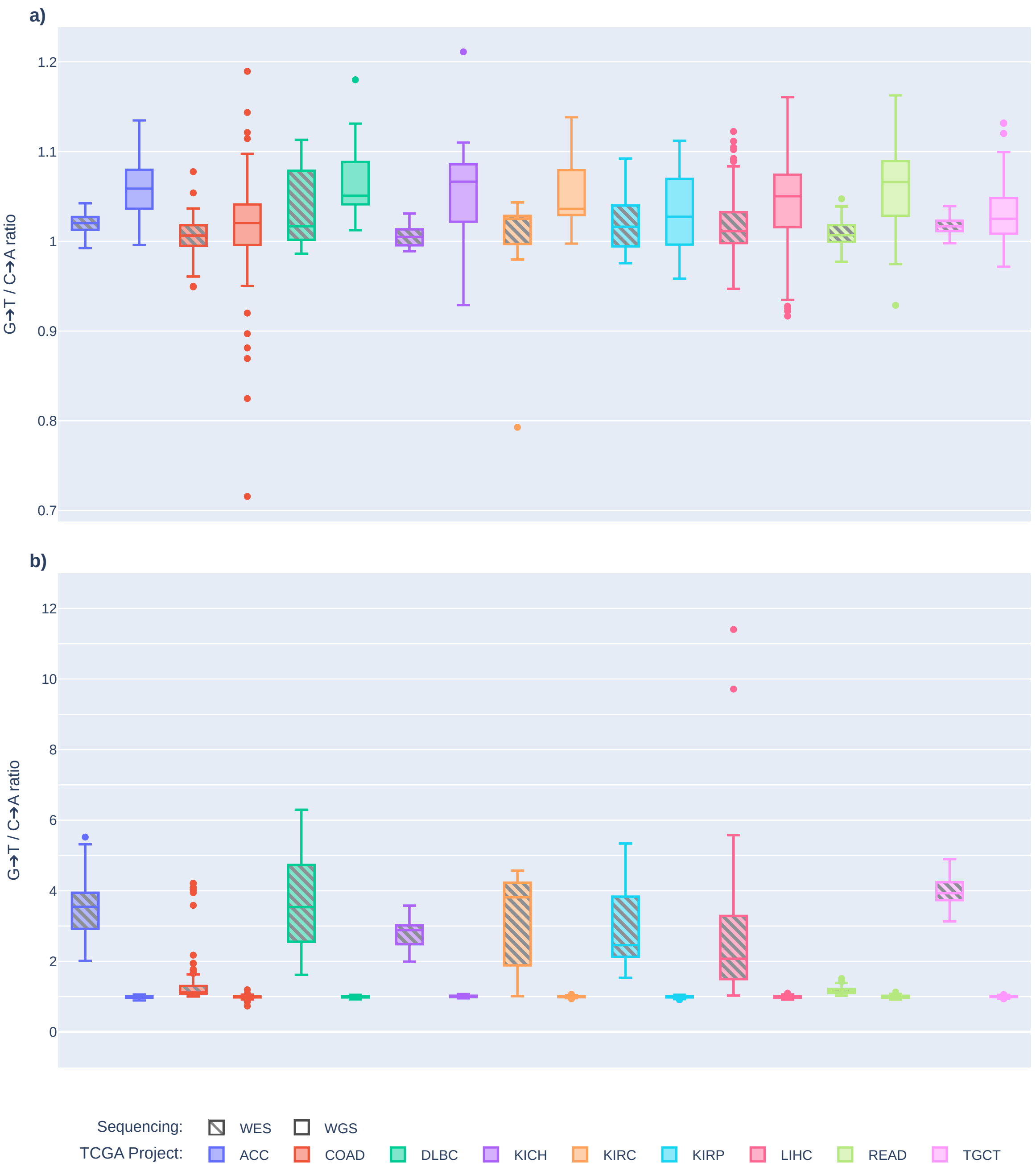

Fig. S6. G>T versus C>A mismatch asymmetry in TCGA WGS versus TCGA WES. The distribution of G>T versus C>A mismatch asymmetry ratios of each of nine TCGA cohorts, relative to the **a)** transcription strand and **b)** reference strand. WES (striped fill) and WGS (solid fill) samples are shown. WGS samples are subset to chromosome 21 only. G>T and C>A mismatch counts are normalised by the relevant nucleotide content of the captured exome (subset to chromosome 21 in the case of the WGS samples).

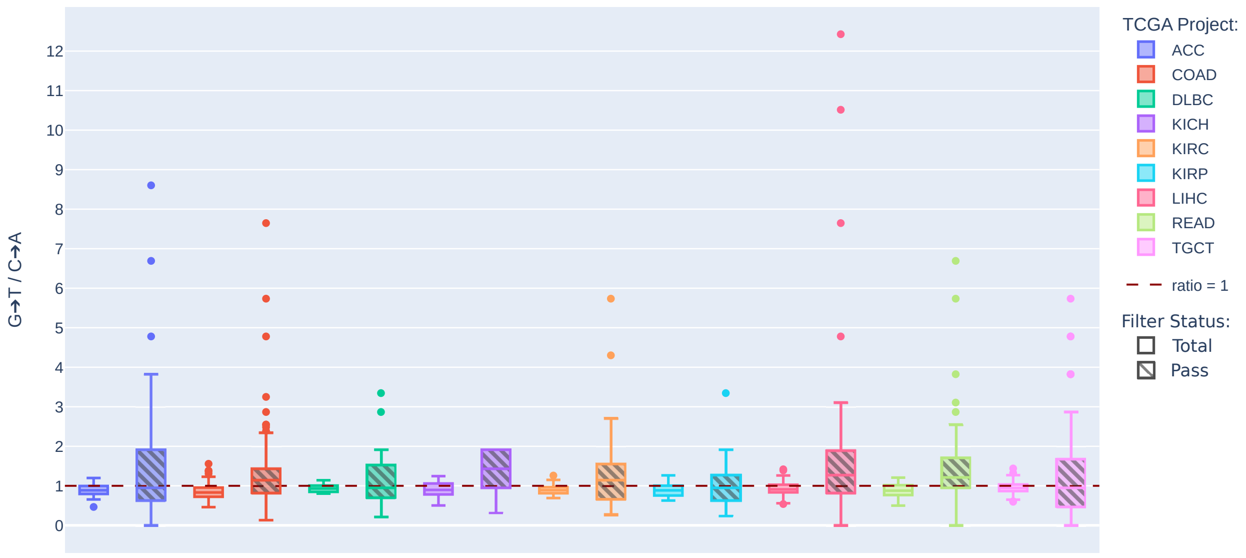

Fig. S7. G>T/C>A variant call ratios by transcription strand in TCGA WES. The distribution of G>T versus C>A variant call ratios, relative to the transcribed strand, for nine TCGA cohorts. Unfiltered (solid fill) and post-filtering (striped fill) variants are shown. The dashed red line indicates the ratio = 1 line, corresponding to equal presence of G>T and C>A variants.

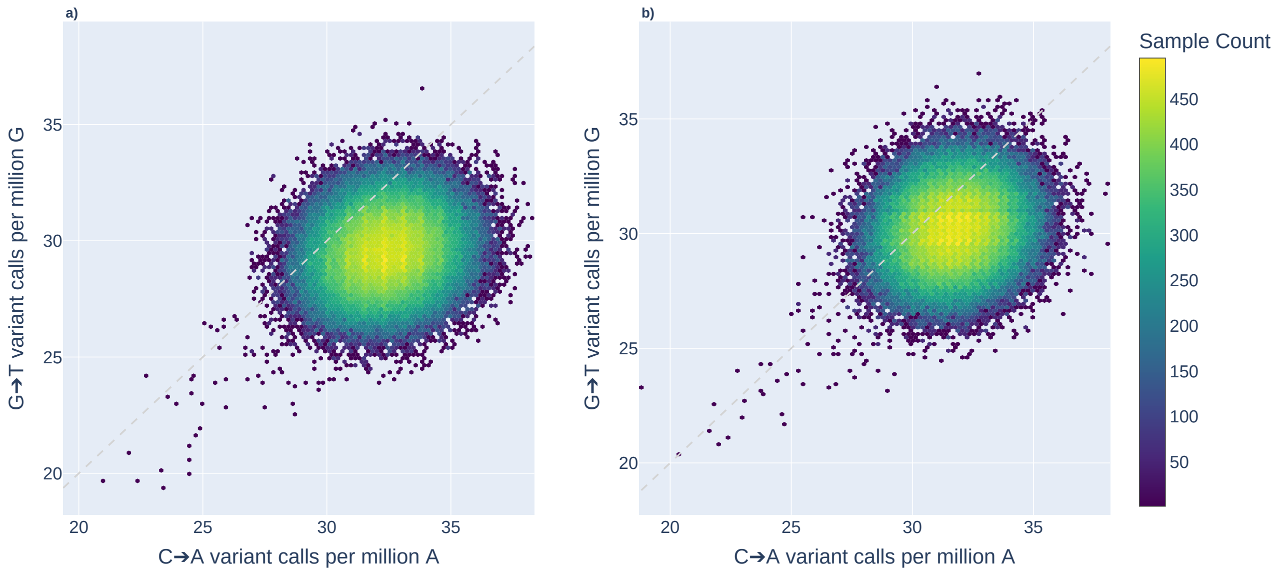

Fig. S8. G>T versus C>A variant calls in UKB WES. Samples are shown binned by hexagon with bin count shown by colour. Variant calls have common SNP loci removed, and include only heterozygous G>T/C>A calls relative to the **a)** reference strand and **b)** transcribed strand. The dashed y = x line represents equal numbers of G>T and C>A calls. Variant counts are normalised by the number of nucleotides in the captured exome.
